# Impact of magnetic resonance-guided versus conventional radiotherapy workflows on organ at risk doses in stereotactic body radiotherapy for lymph node oligometastases

**DOI:** 10.1016/j.phro.2022.06.011

**Published:** 2022-06-30

**Authors:** Anita M. Werensteijn-Honingh, Petra S. Kroon, Dennis Winkel, J. Carlijn van Gaal, Jochem Hes, Louk M.W. Snoeren, Jaleesa K. Timmer, Christiaan C.P. Mout, Gijsbert H. Bol, Alexis N. Kotte, Wietse S.C. Eppinga, Martijn Intven, Bas W. Raaymakers, Ina M. Jürgenliemk-Schulz

**Affiliations:** Department of Radiotherapy, University Medical Center Utrecht, Utrecht, The Netherlands

**Keywords:** Bowel, Duodenum, MR-linac, CBCT-linac, Stereotactic body radiotherapy, Lymph node metastases

## Abstract

**Background and purpose:**

Magnetic resonance (MR)-linac delivery is expected to improve organ at risk (OAR) sparing. In this study, OAR doses were compared for online adaptive MR-linac treatments and conventional cone beam computed tomography (CBCT)-linac radiotherapy, taking into account differences in clinical workflows, especially longer session times for MR-linac delivery.

**Materials and methods:**

For 25 patients with pelvic/abdominal lymph node oligometastases, OAR doses were calculated for clinical pre-treatment and daily optimized 1.5 T MR-linac treatment plans (5 × 7 Gy) and compared with simulated CBCT-linac plans for the pre-treatment and online anatomical situation. Bowelbag and duodenum were re-contoured on MR-imaging acquired before, during and after each treatment session. OAR hard constraint violations, D_0.5cc_ and D_10cc_ values were evaluated, focusing on bowelbag and duodenum.

**Results:**

Overall, hard constraints for all OAR were violated less often in daily online MR-linac treatment plans compared with CBCT-linac: in 5% versus 22% of fractions, respectively. D_0.5cc_ and D_10cc_ values did not differ significantly. When taking treatment duration and intrafraction motion into account, estimated delivered doses to bowelbag and duodenum were lower with CBCT-linac if identical planning target volume (PTV) margins were used for both modalities. When reduced PTV margins were achievable with MR-linac treatment, bowelbag doses were lower compared with CBCT-linac.

**Conclusions:**

Compared with CBCT-linac treatments, the online adaptive MR-linac approach resulted in fewer hard planning constraint violations compared with single-plan CBCT-linac delivery. With respect to other bowelbag/duodenum dose-volume parameters, the longer duration of MR-linac treatment sessions negatively impacts the potential dosimetric benefit of daily adaptive treatment planning.

## Highlights

• Fewer hard organ at risk constraints exceeded with an magnetic resonance (MR)-linac.
• Bowelbag dose lower with MR-linac if planning target volume (PTV) margins reduced.
• If identical PTV margins, no dosimetric advantage MR-linac for bowelbag or duodenum.
• Longer online workflow time on MR-linac decreases dosimetric advantages of MR-linac.

## 1 Introduction

Clinical implementation of magnetic resonance (MR)-guided radiotherapy is rapidly increasing [1]. The superior soft-tissue contrast of MR imaging (MRI) compared with cone beam computed tomography (CBCT) allows improved visualization of target volumes and nearby organs at risk (OAR) [2]. With an MR-linac, MRI scans acquired before, during and after radiation dose delivery are used to optimize the treatment plan for each session [3], [4]. As a result of daily contour adaptation, online plan optimization and longer dose delivery times, treatment sessions using MR-linac take roughly 20–40 min longer compared with CBCT-linac [1]. The longer session duration with MR-linac puts time pressure on all of these steps and as such implies a trade-off between plan optimization time and overall session time [4].

Dosimetric comparisons from two ‘in silico’ studies and from treatment plans for actual online MR-guided delivery indicate an advantage of MR-guided online adaptive radiotherapy for SBRT treatment of patients with (lymph node) oligometastases [5], [6], [7], [8]. Fewer OAR constraint violations and lower mean OAR doses were reported using MR-guided online adaptive delivery compared with non-adaptive or CBCT-linac delivery [5], [6], [7]. Target coverage was also improved with MR-guided delivery, for patients with multiple targets or in the general population of abdominal/thoracic targets [5], [7], [8]. However, not all dosimetric evaluations of clinically delivered MR-guided radiotherapy showed a benefit compared with non-adaptive delivery: for prostate radiotherapy, both MR-linac and CBCT-linac delivery were estimated to achieve 98% of the OAR planning constraints, and better dosimetric outcomes on MR-linac were only seen for patients with an OAR close to the target [9]. For liver SBRT, online adaptive treatment with an MR-linac improved PTV coverage and OAR sparing only in case of OAR within 2 cm of the PTV, which comprised 53% of the population. On the contrary, 47% of the patients included in the study did not benefit from daily MR-guided plan optimization [10]. PTV coverage during treatment of patients with single lymph node oligometastases was excellent with both MR-linac and CBCT-linac delivery [8]. Furthermore, the longer duration of MR-linac treatment sessions has not been taken into account in most of the above-mentioned studies, which may have resulted in an overestimation of the dosimetric improvements using MR-guided radiotherapy [11].

The purpose of this study was to evaluate OAR doses during the first 19 months of online adaptive 1.5 T MR-linac treatment for patients with lymph node oligometastases and compare with data from simulated CBCT-linac delivery. Differences in the currently available clinical workflows for MR-linac and CBCT-linac delivery were taken into account, such as session duration and the associated OAR intrafraction motion.

## 2 Materials and methods

### 2.1 Patients and MR-linac treatment

For this study we selected 25 patients: the first 15 and 10 patients with pelvic and abdominal lymph node targets, respectively, for whom specific OAR had been used for daily online plan optimization on a 1.5 T Unity MR-linac (Elekta AB, Stockholm, Sweden) [12]. For patients with pelvic targets, either bowelbag, rectum or bladder had to have been taken into account, and duodenum for patients with abdominal targets. All patients gave written informed consent for use of their clinical and technical data as part of an IRB-approved observational study (www.trialregister.nl/trial/9252). The RATING score for treatment plan comparison of this study was 93% (187 of 201, Supplementary Table 3) [13].

All patients were treated with a prescribed dose of 5 times 7 Gy to 95% of the PTV(s) in a single treatment plan, with D_0.1cc_ < 47.25 Gy (135% of prescribed dose). An offline pre-treatment intensity modulated radiotherapy (IMRT) plan with 6–10 beam angles was created after image fusion of MRI and PET/CT scans with the planning CT scan. GTVs consisted of target lymph nodes; 3 mm PTV margins were applied [14]. OAR planning constraints are shown in Supplementary Table 1. Patients were immobilized using a vacuum cushion (BlueBAG BodyFIX, Elekta AB), with the exception of 4 patients with pelvic targets [15]. Patients with mesenteric or high para-aortic targets (above the renal veins) were treated whilst wearing a custom fitted polyurethane Neofrakt abdominal corset (Spronken Orthopedie NV, Genk, Belgium) [16]. For each fraction, MRI scans were acquired before, during and after radiation delivery. MRI scans used for this study included a transverse 3D T1-weighted FFE scan and a transverse 3D T2-weighted TSE scan [15]. The adapt to shape workflow was used, with daily contour adaptation and plan optimization using a predefined template for treatment planning [8], [12]. Contours of target lymph nodes (GTVs) and OAR within 2 cm of PTVs were deformed and manually adapted. An optimized IMRT treatment plan was created for each fraction, OAR planning constraints were prioritized above PTV coverage [8], [12]. The average ‘on couch time’ (time between the start of the session (first MRI scan) and the end of radiation delivery) of the complete workflow is 32 min [12], [15]. Further details regarding treatment plan generation are provided in the Supplementary Material.

### 2.2 Simulation of CBCT-linac treatment

10 MV Agility CBCT-linac (Elekta AB) SBRT was simulated by creating Volumetric-Modulated Arc Therapy (VMAT) plans for each patient. VMAT dual arc beams were used, with arc length of 180-360° depending on location and number of lesions. The treating radiation oncologist determined target visibility on CBCT, if a CBCT scan was not available this decision was based on target appearance on the CT scan, taking target location and the physician’s clinical expertise with CBCT-guided SBRT treatments into account. According to our clinical practice, PTV margin was 3 mm but larger PTV margins (5–8 mm) were used in case of poor target visibility on CBCT or in some cases with multiple targets to compensate for interfraction motion and rotations [17]. These treatment plans will be referred to as ‘CBCT-linac with individualized margin’. To investigate the influence of PTV margin reduction, another set of CBCT-linac plans was created with 3 mm PTV margins for all cases: ‘CBCT-linac with 3 mm margin’.

Daily MR-linac contours were used for recalculating the VMAT plans on daily anatomy: electron density information was retained by matching and deforming initial planning CT to the daily MRI data. An optimal online translation was simulated by assuming the correction reference point for single lymph nodes to be equal to the isocenter and by placing it in the center of the daily GTV contour [8]. For patients with multiple PTVs, the isocenter was placed in the center of one of the PTVs or between the PTVs, depending on the choice of the radiation oncologist. In case of two separate CBCT-linac plans, doses were summed.

### 2.3 Offline contouring of OAR of interest

For all MR-linac treatment fractions, we performed offline re-contouring of specific OAR: bowelbag, rectum and bladder for patients with pelvic targets and duodenum for patients with abdominal targets. OAR were contoured on transversal slices within a cranial-caudal extent of PTV(s) + 2 cm, on MRI scans that were obtained at the start of each fraction, at the time of position verification (PV) and directly after radiation delivery. For this study bowelbag was defined as the outer contours of small and large bowel loops and included the sigmoid colon, starting at the recto-sigmoid junction. Multiple observers contributed to OAR contouring under supervision of radiation oncologists.

### 2.4 Time points for dosimetric comparison of MR-linac and CBCT-linac treatment

Radiation doses received by OAR were investigated at three time points. First, the ‘offline pre-treatment’ plans were compared, using clinical target and OAR contours from offline pre-treatment imaging. Secondly, the ‘daily plan’ time point was based on MRI scans acquired at the start of each treatment session with (adapted) online contours. Finally, the ‘estimated delivered’ dose was calculated at the time point roughly halfway through radiation delivery, taking into account approximated session durations with MR-linac and CBCT-linac delivery based on previous experience [15]. For MR-linac, linear interpolation was used between dosimetric results based on PV and post-delivery scans. For CBCT-linac, dosimetric results were interpolated between daily plan and PV scans.

### 2.5 Dose-volume histogram parameters

Dose received by OAR was investigated with two main dose-volume histogram (DVH) parameters: maximum dose received by 0.5 and 10 cc, 1 cc = 1 cm^3^, of the OAR (D_0.5cc_ and D_10cc_). D_1cc_, D_2cc_, D_5cc_, D_9cc_, and OAR volumes that received 15–35 Gy (V_15Gy_, V_20Gy_, V_25Gy_, V_30Gy_, V_35Gy_) were also calculated. An in-house developed software package was used to determine DVH parameters [18]. DVH parameters were averaged over the five treatment sessions for each patient. Violations of OAR planning constraints (Supplementary Table 1) were calculated for individual treatment fractions.

### 2.6 SBRT plan quality metrics

To investigate differences in SBRT plan quality between MR-linac and CBCT-linac plans, offline pre-treatment plans were compared using four dedicated metrics from the NRG-BR001 phase 1 trial [19], [20]:

(1). Homogeneity index (HI) = PD*/D_max_, acceptable if 60% ≤ HI ≤ 90%,
  with actual prescription dose (PD*) defined as dose received by 95% of the PTV(s)
(2). Volume ratio of PD* isodose to PTV (R_100%_) = V_PD*_/V_PTV_, acceptable if R_100%_ ≤ 1.5, preferred R_100%_ < 1.2,
(3). Volume ratio of 50% PD* isodose to PTV (R_50%_) = V_(PD*/2)_/V_PTV_,
(4). D_2cm_ = max. dose at 2 cm from PTV/PD*,
  with limits for acceptable/preferred values for R_50%_ and D_2cm_ that depend on PTV (Supplementary Table 2).

### 2.7 Statistics

The open source R software package (v 4.1.0) was used (R Foundation for Statistical Computing, Vienna, Austria; http://www.R-project.org/). Two-sided Wilcoxon signed-rank tests were used to test for statistically significant differences in D_0.5cc_, D_1cc_, D_2cc_, D_5cc_, D_9cc_ and D_10cc_ between MR-linac and both CBCT-linac plans; p < 0.05 was considered significant.

## 3 Results

A total of 25 patients with 1–3 pelvic and/or abdominal lymph node oligometastases who were treated between August 2018 and February 2020 were included in this study. Patient characteristics are shown in Table 1; for GTV locations see Supplementary Fig. 1.

**Table 1:** Patient characteristics (N = 25). Anatomical locations of the GTV(s) are specified as pelvic (caudal of aortic bifurcation) or para-aortic/mesenteric (cranial of aortic bifurcation), this is shown in more detail in Supplementary Fig. 1. N: number; GTV: gross tumor volume; PTV: planning target volume; ECOG: Eastern Cooperative Oncology Group. PTV margins are shown for the simulated CBCT-linac treatment, in case of multiple PTV margins for a patient the largest margin is reported; 3 mm PTV margins were used for the clinical MR-linac treatment for all patients.

Characteristic		N patients
Location	Pelvic	15
	Para-aortic/mesenteric	10

N GTVs	1	17
	2	4
	3	4

N PTVs	1	18
	2	6
	3	1

GTV in cc (mean (sd))	Mean of GTVs per patient	6.6 (11.6)
	Sum of GTVs per patient	7.8 (14.3)

PTV in cc (mean (sd))	Mean of PTVs per patient	16.6 (27.1)
	Sum of PTVs per patient	17.5 (26.7)

PTV margin for CBCT-linacTreatment in mm	3	11
	5	8
	8	6

Treatment plans per patientFor CBCT-linac treatment	1	23
	2	2

Primary tumor	Prostate	16
	Colorectal	6
	Esophageal	1
	Lung	1
	Hepatocellular carcinoma	1

ECOG performance status	0	16
	1	8
	2	1

**Fig. 1:**
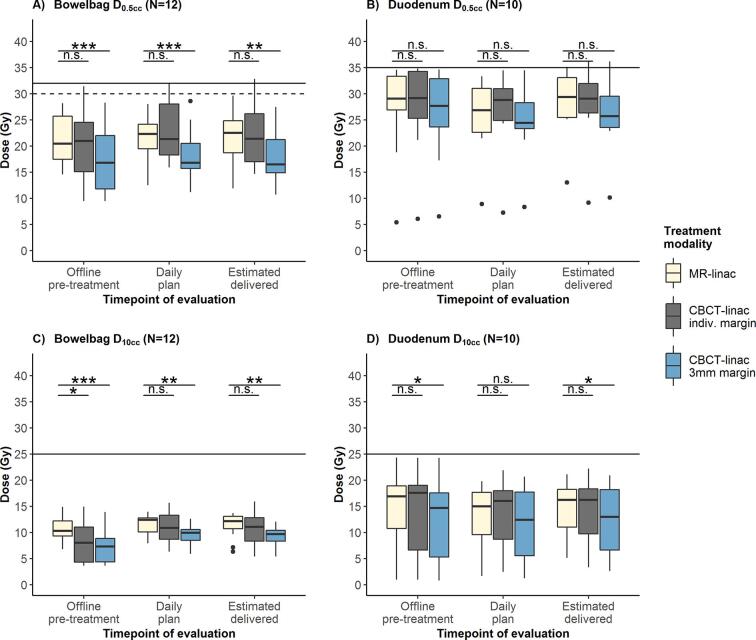
Comparison of bowelbag and duodenum dose using MR-linac and CBCT-linac SBRT for lymph node oligometastases. D_0.5cc_ and D_10cc_ were calculated for three time points: offline pretreatment anatomy (offline pretreatment), anatomy at the start of each treatment fraction (daily plan) and estimated anatomy at the moment of radiation delivery for each fraction (estimated delivered, average of pre/PV scans for CBCT-linac and average of PV/post scans for MR-linac). Averages per patient are shown for MR-linac (3 mm PTV margin), CBCT-linac with the individualized PTV margin and CBCT-linac with 3 mm PTV margin. Center line indicates median, hinges depict 25th and 75th percentiles (inter-quartile range, IQR) and whiskers extend from the hinge to the largest/smallest value at maximally 1.5*IQR. Outlying data points (beyond end of the whiskers) are plotted individually. Hard constraints are plotted as solid lines, soft constraints as dashed lines. Asterisks depict significant differences in DVH parameters between MR-linac and both CBCT-linac plans (Mann-Whitney *U* test (two-sided), n.s. p ≥ 0.05, *p < 0.05, **p < 0.01, ***p < 0.001), with the lower bars indicating differences between MR-linac and CBCT-linac with the individualized PTV margins, and the upper bars indicating differences between MR-linac and CBCT-linac plans with 3 mm PTV margins.

OAR doses in the pre-treatment plans were similar when comparing MR-linac plans with the CBCT-linac plans with individualized PTV margins, dosimetric outcomes are shown for bowelbag and duodenum (Fig. 1, Supplementary Fig. 2). Bowelbag doses were significantly lower for CBCT-linac plans with 3 mm margins, differences were smaller and mostly non-significant for duodenum (Fig. 1, Supplementary Fig. 2). SBRT plan quality metrics indicated that CBCT-linac plans with 3 mm PTV margins were more conformal than MR-linac plans (Supplementary Fig. 3).

**Fig. 2:**
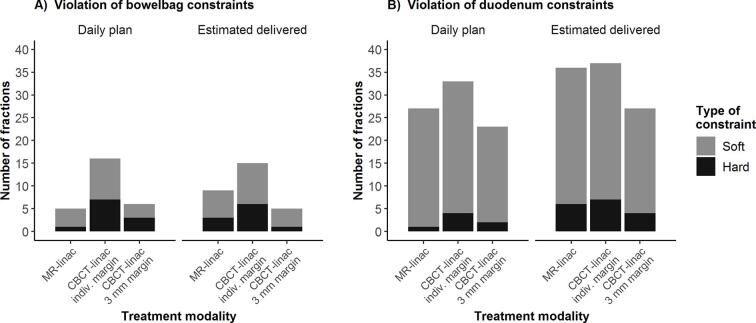
Violation of planning constraints using MR-linac and CBCT-linac SBRT for lymph node oligometastases. Number of individual treatment fractions for which soft and hard constraints were violated are shown for bowelbag (A) and duodenum (B). Results were calculated for MR-linac (3 mm PTV margin), CBCT-linac with the individualized PTV margin and CBCT-linac with 3 mm PTV margin. Constraint violations are shown at two time points: anatomy at the start of the treatment fraction (daily plan) and estimated anatomy at the moment of radiation delivery (estimated delivered, average of pre/PV scans for CBCT-linac and average of PV/post scans for MR-linac). Applicable planning constraints are shown in Supplementary Table 1.

**Fig. 3:**
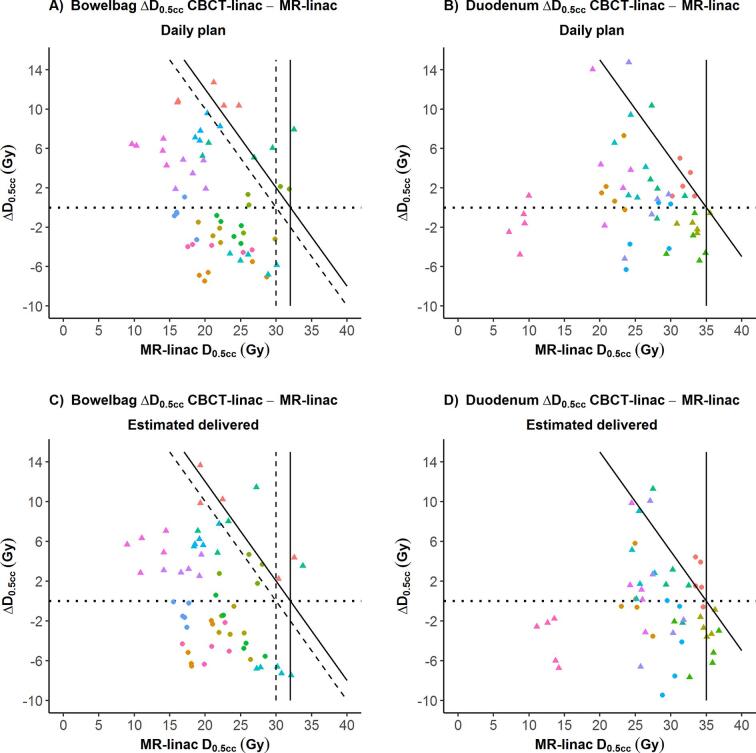
Differences in bowelbag and duodenum dose using MR-linac and CBCT-linac SBRT for lymph node oligometastases. Differences in D_0.5cc_ (ΔD_0.5cc_) between MR-linac and CBCT-linac, both with 3 mm PTV margins, are shown for bowelbag (A and C) and duodenum (B and D), at daily plan time point (A and B) or estimated delivered time point (C and D). Differences are plotted for each individual fraction, colors represent the patients (N = 12 for bowelbag and N = 10 for duodenum). Dots represent patients with a 3 mm PTV margin for CBCT-linac, triangles indicate patients with a CBCT-linac PTV margin of 5 mm or larger. MR-linac PTV margin was always 3 mm. ΔD_0.5cc_ = 0 Gy is visualized with a dotted horizontal line. A negative ΔD_0.5cc_ indicates a lower D_0.5cc_ using CBCT-linac compared with MR-linac. Hard constraints are plotted as solid lines, soft constraints as dashed lines. Constraints are plotted both vertically and diagonally: dots to the right of a vertical line indicate fractions for which the constraint was violated with MR-linac, dots to the upper-right of a diagonal line indicate fractions with a constraint violation using CBCT-linac.

For the actual treatment sessions, daily optimized MR-linac plans were compared with CBCT-linac plans. When considering all applicable OAR constraints (Supplementary Table 1), hard constraints were violated in 6/125 treatment fractions (5%) of the daily optimized MR-linac treatment plans (‘daily plan’ time point). For CBCT-linac plans with individualized and 3 mm margins, OAR constraints were violated in 28/125 sessions (22%) and in 16/125 (13%), respectively. Bowelbag and duodenum constraint violations are shown in Fig. 2; rectum and bladder constraint violations were not observed (data not shown). The largest constraint violation was observed for CBCT-linac with bowelbag D_0.5cc_ being 40.4 Gy for a single fraction (hard constraint <32 Gy). Bowelbag and duodenum D_0.5cc_ and D_10cc_ were not significantly different between MR-linac and CBCT-linac with individualized margins (Fig. 1). When comparing MR-linac and CBCT-linac treatment plans with 3 mm PTV margins, bowelbag doses were significantly lower for CBCT-linac plans; no significant differences were observed for duodenum (Fig. 1, Supplementary Fig. 2). Differences in bowelbag D_0.5cc_ between MR-linac and CBCT-linac appeared to be related to PTV margins: when larger PTV margins were used for CBCT-linac, bowelbag doses were lower using MR-linac in 6 out of 7 cases. In case of identical PTV margins for both modalities, bowelbag doses were lower using CBCT-linac in 7 out of 8 cases (Fig. 3).

Furthermore, we estimated the doses that were actually delivered to OAR, taking intrafraction motion into account (‘estimated delivered’ time point). Bowelbag or duodenum hard constraints would have been violated in 9, 13 and 5 treatment sessions using MR-linac, CBCT-linac with individualized margins and CBCT-linac with 3 mm margins, respectively (Fig. 2). When averaged (per case) over the five treatment sessions, maximum violations of hard constraints were 0.8 and 1.2 Gy for bowelbag and duodenum, respectively (Fig. 1). Rectum and bladder constraint adherence was 100% with both modalities (data not shown). No significant differences were observed for bowelbag and duodenum D_0.5cc_ and D_10cc_ when comparing MR-linac and CBCT-linac with individualized PTV margins (Fig. 1). When comparing MR-linac with CBCT-linac with 3 mm PTV margins, all tested DVH parameters for bowelbag and duodenum were significantly lower using CBCT-linac delivery except duodenum D_0.5cc_ (Fig. 1).

**Fig. 4:**
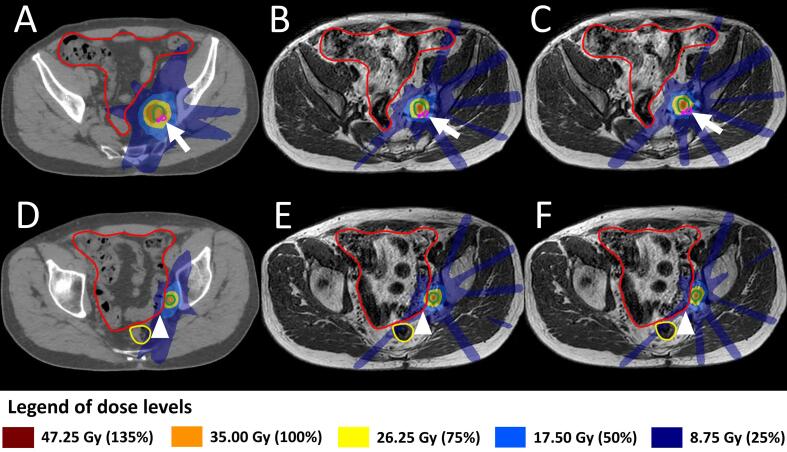
Potential for improvement of the online plan optimization during MRgRT on a 1.5 T MR-linac. Treatment plans at the daily plan time point are shown for an illustrative case (patient 19, fraction 5): the CBCT-linac plan with individualized margins (A and D), the clinically delivered MR-linac plan (B and E) and an offline re-optimized MR-linac plan (C and F). This patient had three GTVs in two PTVs, with a 3 mm PTV margin for PTV1 and an 8 mm PTV margin for PTV2, with the isocenter placed in PTV1. The daily online MR-linac PTV2 contour (using 3 mm PTV margins used on MR-linac) is shown in green. PTV and OAR hard planning constraints were met for the MR-linac plans, whereas the sacral plexus (pink contour) constraint was violated on the CBCT-linac plan (A, B andC, arrows). Still, the bowelbag (red contour) D_0.5cc_ was 5.8 Gy lower using CBCT-linac (D, E and F, arrowheads). The dose received by the bowelbag could have been further reduced for the clinically delivered MR-linac plan with adjustment of the bowelbag isoeffect settings during online plan optimization, resulting in a plan that met all planning goals, with a lower bowelbag dose (C and F).

In Fig. 4, treatment plans are shown for a case in which the distance between the target and sacral plexus was less in the online treatment situation compared with the pre-treatment anatomy. In this situation the bowelbag D_0.5cc_ was lower using CBCT-linac, despite larger PTV margins. However, the CBCT-linac plan would have violated the sacral plexus D_0.1cc_ with 1 Gy (hard constraint <32 Gy). The MR-linac plan complied with all target and hard OAR constraints, but the bowelbag D_0.5cc_ was 5.8 Gy higher than on the CBCT-linac plan. With some more attention on bowelbag sparing during online plan optimization, the bowelbag D_0.5cc_ could have been reduced with 8.5 Gy without compromising PTV coverage or violating the sacral plexus constraint, as was shown with an offline-optimized plan (Fig. 4).

## 4 Discussion

This is one of the first studies comparing OAR dose parameters of clinically used online MR-linac and simulated CBCT-linac plans. An important difference between MR-linac and CBCT-linac workflows is the duration of treatment sessions. For MR-linac, session duration is longer due to daily online plan optimization and longer dose delivery. Therefore, the impact of intrafraction motion on OAR doses should be considered. Our analysis demonstrates that hard OAR planning constraints were violated less frequently with daily online adaptive MR-linac treatment plans. For other OAR dose-volume parameters, MR-linac treatment provided improved bowelbag sparing when smaller PTV margins were applied compared with CBCT-linac [6]. However, when applying 3 mm PTV margins for both modalities, the ‘estimated delivered’ doses to bowelbag and duodenum were significantly lower with CBCT-linac treatment. This effect may be partially due to our choices regarding daily plan adaptation methodology for MR-linac treatments: first, due to time pressure on daily plan optimization, currently only high-dose DVH parameters are being taken into account and re-contouring of OAR is limited to OAR within 2 cm of the PTV(s). Secondly, the individual pre-treatment plans for the 1.5 T MR-linac, which are used as templates for online plan adaptation, have to allow for fast online plan adaptation, must prevent unnecessary compromise of target dose, and might therefore be somewhat less conformal than the CBCT pre-treatment plans [9]. Finally, intrafraction OAR motion is not corrected for in our current clinical MR-linac workflow despite the longer time needed for dose delivery. Thus, specific aspects of the MR-guided online adaptive treatment workflow are likely to have contributed to our finding of fewer than anticipated benefits of current 1.5 T MR-linac delivery regarding OAR dosimetry.

A comparison between 1.5 T MR-linac and CBCT-linac dose delivery has previously been reported by Dunlop et al. for prostate radiotherapy (20 times 3 Gy) [9]. They showed that target coverage could be improved using an MR-linac compared with CBCT-linac for patients with OAR close to the target volumes on offline pre-treatment imaging. Adherence to OAR planning constraints was excellent with both modalities. Higher rectum and bladder doses were described for particular cases using MR-linac, consistent with our findings. Henke et al. have reported on online adaptive MR-guided radiotherapy for oligometastatic or unresectable primary abdominal malignancies with a fractionation scheme of 5 times 10 Gy [7]. OAR planning constraint violations were observed for 63% of fractions, mainly for small bowel, duodenum and stomach. Target dose could be escalated in 21% of fractions. The benefits from online adaptive MR-guided radiotherapy seem to have been larger for this specific fractionation scheme. However, in both aforementioned studies the “estimated delivered doses” had not been calculated. With our current fractionation scheme of five SBRT fractions of 7 Gy, the estimated OAR constraint violations for individual treatment fractions on CBCT-linac would have largely evened out over the course of treatment, with a maximum violation of bowelbag and duodenum D_0.5cc_ constraints of 1.2 Gy. Stricter adherence to OAR planning constraints may be important when considering further hypofractionation [21].

In addition to daily plan adaptation, MR-linac treatment also enables offline reconstruction of OAR doses from previous treatment sessions using MRI scans acquired during and after radiation delivery. The planning goals for following fractions can thus be adapted based on OAR doses estimated to have been delivered during previous fractions. Such a process of dose reconstruction is labor-intensive, but it can be of additional value for patients with an OAR located close to the GTV and for patients with a higher risk of toxicity because of previous radiotherapy or surgery in the target area [22]. Future developments are expected to improve the advantages of MR-linac treatments, such as fast intrafraction plan adaptation [23]. With intrafraction plan adaptation, changes in both target and OAR anatomy during radiation delivery could be incorporated. Finally, we observed a learning curve in our clinical experience regarding MR-linac treatments, with room for improving the workflow and the planning templates. As is shown in Fig. 4, OAR doses can only be ‘as low as reasonably achievable’ (ALARA) with specific attention on reducing OAR doses during online plan optimization, rather than only examining OAR hard constraint adherence. Also, templates for online plan optimization could be improved with addition of other dose-volume parameters, as long as the online plan optimization time remains acceptable [24], [25].

A strength of this study is that OAR have been re-contoured on MRI scans acquired before, during and after each treatment session, which enabled us to estimate the delivered doses to bowelbag and duodenum at realistic time points. A limitation of this study is the application of 3–8 mm PTV margins for CBCT-linac simulations, with 5–8 mm margins in case of poor target visibility on CBCT. These margins reflect our clinical practice but are larger than the 3–5 mm margins that are also commonly used [26], [27]. Furthermore, linear interpolation between MRI scans was used to estimate delivered OAR doses, which may have disregarded potential OAR motion between the acquisition of MRI scans.

Compared with CBCT-linac treatments, the online adaptive MR-linac approach resulted in fewer hard planning constraint violations compared with single-plan CBCT-linac delivery. With respect to other dose-volume parameters for bowelbag and duodenum, differences in OAR sparing depended on the treatment margins. MR-linac workflow aspects, such as longer treatment sessions, limited time for online plan optimization and the absence of compensation for OAR intrafraction motion, currently seem to decrease the potential advantages of online adaptive MR-guided delivery.

## Funding

This work was supported by the Dutch Cancer Society under Grant 2015-0848.

## Declaration of Competing Interest

The authors declare the following financial interests/personal relationships which may be considered as potential competing interests: The overarching University Medical Center Utrecht MR-linac scientific project, including employment of multiple authors, has been partly funded by Elekta AB (Stockholm, Sweden). Elekta did not have any part in the design, execution or analysis of this study. The authors declared that there is no other conflict of interest.
