# Supplementary material for: Impact of magnetic resonance-guided versus conventional radiotherapy workflows on organ at risk doses in stereotactic body radiotherapy for lymph node oligometastases
Source: Phys Imaging Radiat Oncol. 2022 Jun 30;23:66–73. doi: 10.1016/j.phro.2022.06.011 (PMC9263510; doi:10.1016/j.phro.2022.06.011)
Supplement: Supplementary data 1 [file mmc1.pdf]

Supplementary Material for Werensteijn-Honingh *et al.*, 2022:

Impact of magnetic resonance-guided versus conventional radiotherapy workflows on organ at risk doses in stereotactic body radiotherapy for lymph node oligometastases

Detailed methods description

Part I: MR-linac treatment plan generation

The clinically delivered MR-linac plans were used. Monaco versions 5.4 and 5.5 (Elekta AB) have been used with a GPUMCD 7 MV FFF photon beam model and with a 1.5 T magnetic field in superior-inferior patient direction. Grid sizes of 2-3 mm were used, with a statistical uncertainty of 3% per control point and with maximally 45 segments per plan, with minimal segment width of 0.5 cm. Beam angles were non-uniformly spaced, avoiding the couch at 115-135° and 225-245° and avoiding the cryostat connection pipe at 8-18°. During online plan optimization, beam angles were identical to the offline pre-treatment plan.

In general, the treatment planning template prioritization was first OAR planning constraints, then GTV coverage, then PTV coverage, then further decreasing the dose to OAR within 2 cm of the PTV, then decreasing the dose in other parts of the ring of 2 cm around the PTV (quadratic overdose cost function) and finally decreasing the dose received by the total body (max dose cost function).

Part II: CBCT-linac treatment plan generation

An experienced RTT created these CBCT-linac plans according to the planning template that is used for CBCT-linac treatments in our clinic. Monaco version 5.11 (Elekta AB) was used, with Monte Carlo dose calculation and with a grid size of 2 mm. The statistical uncertainty was 8% per control point, with maximally 144 control points per arc and with a minimal segment width of 0.5 cm. OAR constraints were identical for both modalities (Supplementary Table 1) and for both modalities OAR planning constraints were prioritized above PTV coverage.

Part III: Statistics

V_{15Gy}, V_{20Gy}, V_{25Gy}, V_{30Gy} and V_{35Gy} were reported without statistical testing to limit the number of comparisons.

Supplementary Table 1. Planning constraints for organs-at-risk in this study, for SBRT delivery in 5 fractions. Constraints that were used during daily online plan adaptation are indicated with an asterisk.

Organ at risk	Parameter	Hard constraint	Soft constraint
Bladder	$D_{0.5cc}$	< 42 Gy*	
	D_{5cc}	< 37 Gy	
Bowelbag / Large bowel	$D_{0.5cc}$	< 32 Gy*	< 30 Gy
	D_{5cc}		< 25 Gy
	D_{10cc}	< 25 Gy	
Duodenum	$D_{0.5cc}$	< 35 Gy*	
	D_{1cc}		< 33 Gy
	D_{5cc}		< 25 Gy
	D_{9cc}		< 15 Gy
	D_{10cc}	< 25 Gy	
Rectum	$D_{0.5cc}$	< 40 Gy*	
	D_{1cc}	< 38 Gy	< 35 Gy
Sacral plexus / Nerve roots / Cauda equina	$D_{0.1cc}$	< 32 Gy*	
	D_{5cc}	< 30 Gy	
Stomach	$D_{0.5cc}$	< 35 Gy*	< 33 Gy
	D_{5cc}		< 25 Gy
	D_{10cc}	< 25 Gy	
Ureter	$D_{0.5cc}$	< 42 Gy*	

Supplementary Table 2. Limits for SBRT plan quality/conformity metrics that depend on PTV volume. Recommended metric values are shown for maximum dose at 2 cm from PTV (D_{2cm}) relative to actual prescription dose (PD*) and for volume ratio of volume receiving 50% of PD* to PTV ($R_{50\%}$), as defined in the NRG-BR001 phase 1 trial [1,2]. Linear interpolation between table entries is required for PTV values not specified. Limits for D_{2cm} indicate acceptable values and for $R_{50\%}$ preferred values. Note that the $R_{50\%}$ was evaluated for summed doses in case of separate treatment plans for CBCT-linac, to allow for comparison with the MR-linac plan; for the NRG-BR001 trial $R_{50\%}$ would have been evaluated separately for each lesion/plan.

PTV (cc)	$R_{50\%}$ (-)	D_{2cm} (%)
< 1.8	< 7.5	< 57
< 3.8	< 6.5	< 57
< 7.4	< 6.0	< 58
< 13.2	< 5.8	< 58
< 22.0	< 5.5	< 63
< 34.0	< 5.3	< 68
< 50.0	< 5.0	< 77
< 70.0	< 4.8	< 86
< 95.0	< 4.4	< 89
< 126.0	< 4.0	< 91
<163.0	< 3.7	< 94

References

1) Al-Hallaq HA, Chmura S, Salama JK, Winter KA, Robinson CG, Pisansky TM, et al. Rationale of technical requirements for NRG-BR001: The first NCI-sponsored trial of SBRT for the treatment of multiple metastases. Pract Radiat Oncol 2016;6:e291-8. https://doi.org/10.1016/j.prro.2016.05.004.
2) Chmura SJ, Winter K, Salama JK, Robinson CG, Pisansky TM, Borges V, et al. Phase I Trial of Stereotactic Body Radiation Therapy (SBRT) to Multiple Metastatic Sites: A NRG Oncology Study. Int J Radiat Oncol Biol Phys 2018;102:s68-9. https://doi.org/10.1016/j.ijrobp.2018.06.187.

Supplementary Table 3. RATING score card [1].

RATING score sheet		Points	Applicable/ relevant	Answer yes
**Questions for the Introduction**				
	The study aim formulated by research questions			
1	Does the study have a concise and precise study aim, defined with a restricted number of interconnected questions?	10		☑
	The motivation for the research questions			
2	Has relevant up to date literature been included to support the need for the current study?	5		☑
3	Does the study address an existing knowledge gap?	10		☑
**Questions for Materials and Methods**				
4	Is the global study design adequate for answering the posed research questions?	10		☑
5	Is the global study design described in sufficient detail for others to interpret and reproduce the results?	5		☑
	Patient cohort			
6	Are the inclusion and exclusion criteria of the patient cohort described?	1	☑	☑
7	Is the clinical patient information of the cohort presented, including disease type, site(s) and clinical staging?	1	☑	☑
8	Is the included number of patients stated, explained and justified?	1	☑	☐
9	Has there been consideration of the need for ethical and/or legal approval for the study and if needed, is there a statement about this?	5		☑
	Imaging procedures			
10	Have the scanning parameters been reported in sufficient detail (image modalities, equipment model, slice thickness, voxel size, patient position (e.g. head first, supine, etc.) etc.)?	1	☑	☐
11	Has the applied immobilisation equipment been described, (e.g. vendor and type, standard settings, etc.) where relevant?	1	☑	☑
	Treatment machine and settings			
12	Have the treatment machine and relevant parameters been described with sufficient detail (model, beam energy, MLC, etc.)?	1	☑	☑
13	Have the monitor unit reference conditions been defined, where relevant?	1	☐	☐
	Definition of targets and OARs			
14	Has GTV definition been described in sufficient detail, with references if possible?	1	☑	☑
			☐	

15	Has CTV definition been described in sufficient detail, with references if possible?	1		☐
16	Has the establishment of PTVs (or alternatively robustness settings) been described in sufficient detail?	1	☑	☑
17	Have PTV sizes in the patient cohort been described?	1	☑	☑
18	Have OAR definitions been described in sufficient detail, with references if possible?	1	☑	☑
19	Have PRV margins been described in sufficient detail, with references if available?	1	☐	☐
	Treatment planning system and dose calculation			
20	Have all applied dose calculation algorithms been described in sufficient detail?	1	☑	☑
21	For any commercial software used, have the manufacturer, algorithms and specific versions been stated?	1	☑	☑
22	Have all relevant user parameters and settings in the TPS been reported, e.g. beams, dose grid, control point spacing?	1	☑	☑
23	Have all volumes been evaluated with the same software/methodology?	1	☑	☑
	Planning aims and optimisation			
24	Are clear planning aims defined, including imposed hard constraints and planning objectives (with or without soft constraints)?	5		☑
25	Has the ranking of planning objectives (priorities) been described?	5		☑
26	Is the dose prescription clearly defined?	10		☑
27	Is there a narrative description of the applied optimisation process, including the handling of all objectives with their ranking?	5		☑
28	If manual intervention during or after optimisation is allowed, has this been described?	1	☑	☐
	Bias mitigation			
29	Have enough study details been provided such that bias issues could be noted?	5		☑
30	Has bias been sufficiently mitigated to reliably answer the posed research question?	10		☑
	Plan acceptability – minor and major protocol deviations			
31	Was the procedure for assessment of plan acceptability well-described?	1	☐	☐
32	Was the procedure for assessment of minor and major protocol deviations well described?	1	☐	☐
	Plan (re-)normalisation for plan comparisons			
33	Has plan (re-)normalisation been described sufficiently?	1	☐	☐

	Dose-volume parameters for plan evaluation and comparison			
34	Have sufficiently comprehensive dose-volume parameters been used for plan evaluations and comparisons?	5		☑
	Population-mean DVHs			
35	Has the algorithm for creating population-mean/median DVHs been reported?	1	☐	☐
36	Have the definitions of confidence intervals been included?	1	☐	☐
	Plan evaluations by clinicians			
37	Have clinicians scored plans to assess quality?	1	☐	☐
38	Were plan comparisons by clinicians blinded?	1	☐	☐
	Predicted tumour control probability and normal tissue complication probabilities for plan evaluation and comparison			
39	Have any applied TCP models been described and referenced?	1	☐	☐
40	Have any applied NTCP models been described and referenced?	1	☐	☐
	Plan deliverability and complexity			
41	Have methods used to assess plan deliverability and complexity been described in sufficient detail?	1	☐	☐
	Composite plan quality metrics			
42	Is there a sufficient basis (e.g. in the literature) for any selected composite plan quality metrics?	1	☑	☑
43	Is there an adequate description of the calculation of the composite plan quality metrics?	1	☑	☑
	Planning and delivery times			
44	Has measurement of planning times been described in sufficient detail?	1	☑	☐
45	Has the establishment of delivery times been described in sufficient detail?	1	☑	☐
	Statistical analysis			
46	Have proper statistical methods been used and described in sufficient detail?	5		☑
47	In case of multiple testing for research questions, has this been handled appropriately?	1	☑	☐
**Questions for Results**				
48	Does the provided data contribute to (at least partly) answering all aspects of the research questions, e.g. plan acceptability, dosimetric quality, deliverability and planning and delivery times?	10		☑
	Dose distribution reporting			
49	Are complete summaries of the dose distributions in the patient cohort provided (low doses, high doses, OARs, PTV, patient, etc.)?	5		☑

50	Are tables and figures optimised to clearly present the results obtained?	1	☑	☑
51	Have the answers to the research questions been illustrated for an example patient by providing dose distributions, DVHs, etc.?	1	☑	☑
	Plan acceptability reporting – minor and major protocol deviations			
52	In case of treatment technique or planning technique comparisons, was plan acceptability reported separately for each technique?	1	☑	☑
53	Has plan acceptability been reported in sufficient detail: how many plans were acceptable, how many were not and for what reasons (e.g. violation of hard constraints, violation of soft constraints, other reasons)?	1	☑	☑
54	Was there adequate reporting of minor and major protocol deviations?	1	☐	☐
	Deliverability and complexity reporting			
55	Has the deliverability of the plans been adequately reported?	1	☑	☐
56	Have plan deliverability and complexity been investigated in sufficient detail in relation to the posed research questions?	1	☑	☑
	Planning and delivery times reporting			
57	Have planning and delivery times been adequately evaluated and reported?	1	☑	☐
	Patient-specific analyses reporting			
58	Is there sufficient description of inter-patient variations in the results presented?	1	☑	☑
59	Have outlier patients been reported and has any exclusion from population analyses been sufficiently motivated and explained?	1	☑	☑
	Statistical reporting			
60	Are the p-values reported appropriately?	1	☑	☑
61	Are there confidence intervals for the appropriate parameters?	1	☐	☐
**Questions for discussions**				
62	Is there an overall interpretation of the data presented in the Results section as to how the posed research questions are answered?	10		☑
	Comparison with literature			
63	Has the study been sufficiently discussed in the context of existing literature?	5		☑
	Clinical and statistical significance			

64	Does the discussion focus on statistically significant results?	1	☑	☑
65	Is the potential clinical significance of the results clearly discussed (assuming practical application would be feasible)?	5		☑
	Clinical applicability of the study			
66	Is future the clinical applicability sufficiently discussed?	1	☑	☑
	Study limitations			
67	Has the impact of the study limitations on the provided answers to the research questions been sufficiently discussed?	10		☑
	Future work			
68	Has the potential future work arising from the study been discussed?	1	☑	☐
**Questions for conclusions**				
69	Do the presented conclusions represent answers to the posed research questions?	5		☑
70	Are the conclusions fully supported by the results?	5		☑
71	Are the conclusions a fair summary of all results?	5		☑
**Questions for supplementary**				
	Supplementary materials			
72	Is the information presented in the supplementary material of sufficient relevance?	1	☑	☑
73	Is the presentation of the included information of sufficient quality, including readability?	1	☑	☑
74	Has sufficient underlying data been made available or a willingness to share data been indicated, within local data sharing restrictions?	5		☐
**RATING remarks**				
75	Is the RATING score added to the manuscript?	5		☑
76	Is the accompanying question table added to the cover letter or the supplementary material?	1	☑	☑

RATING score		93%	
**RATING fraction**	187	of	201

References

1) Hansen CR, Crijns W, Hussein M, Rossi L, Gallego P, Verbakel W, et al. Radiotherapy Treatment plannINg study Guidelines (RATING): A framework for setting up and reporting on scientific treatment planning studies. Radiother Oncol 2020;153:67-78. https://doi.org/10.1016/j.radonc.2020.09.033.

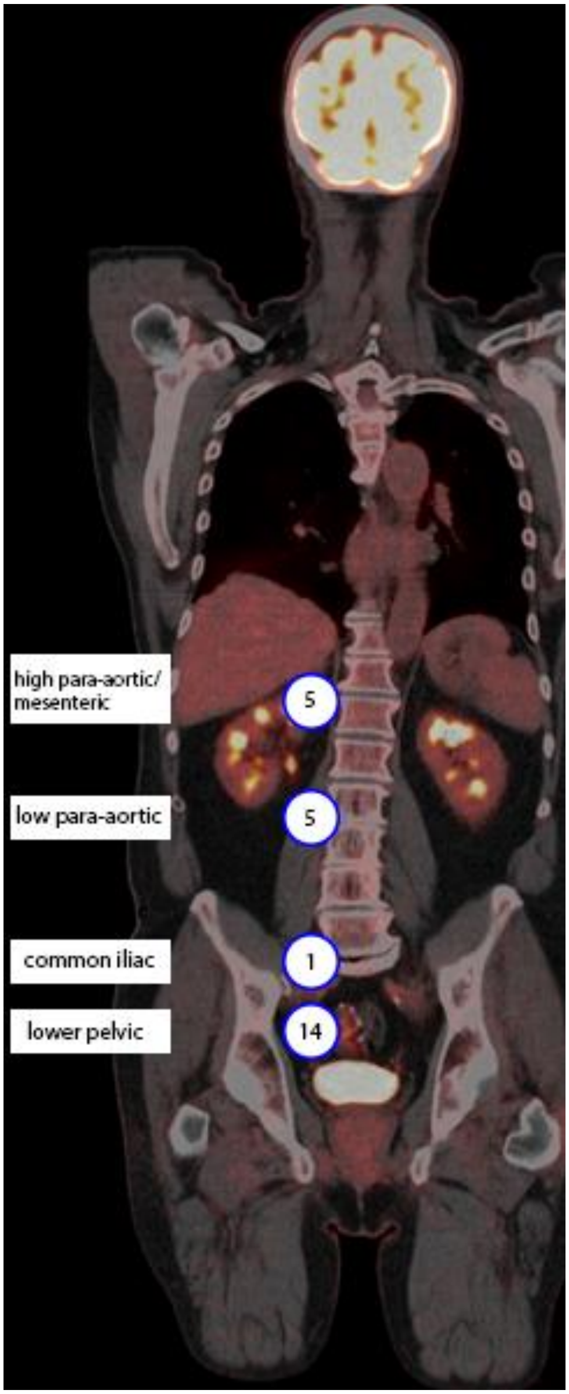

Supplementary Figure 1. Anatomical locations of GTVs for patients in this study. The number of patients is plotted for each anatomical location. For patients with multiple GTVs, the situation was assigned to the location with the most GTVs. Anatomical levels were defined as high para-aortic/mesenteric (cranial of the level of insertion of renal veins into inferior vena cava), low para-aortic (caudal of the renal veins, cranial of aortic bifurcation), common iliac (caudal of aortic bifurcation, cranial of iliac artery bifurcation) and lower pelvic (caudal of iliac artery bifurcation).

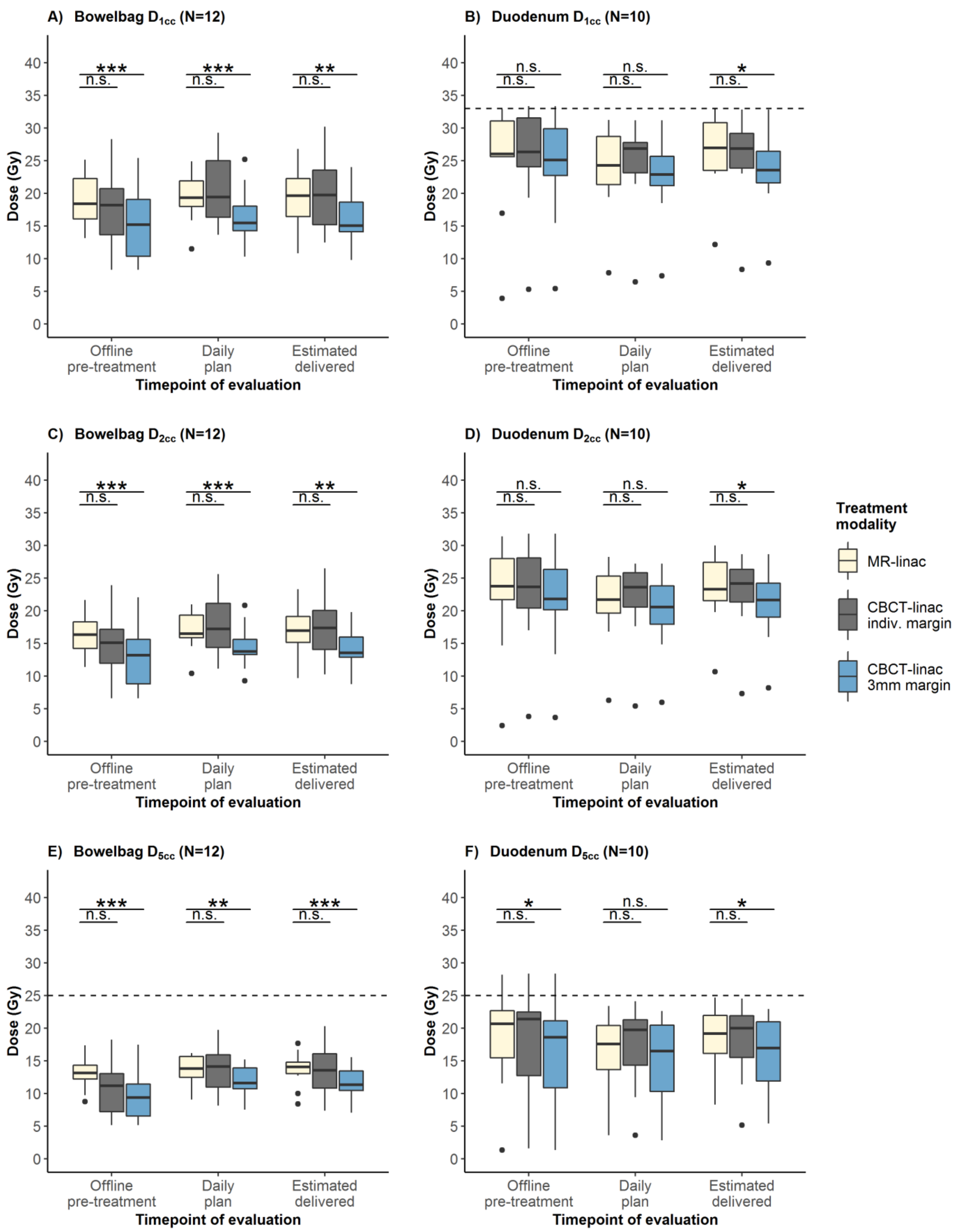

Supplementary Figure 2

Comparison of bowelbag and duodenum dose using MR-linac and CBCT-linac SBRT for lymph node oligometastases. DVH parameters were calculated for three time points: offline pretreatment anatomy

(offline pretreatment), anatomy at the start of each treatment fraction (daily plan) and anatomy at the moment of radiation delivery for each fraction (estimated delivered, average of pre/PV scans for CBCT-linac and average of PV/post scans for MR-linac). Averages per patient are shown for MR-linac (3 mm PTV margin), CBCT-linac with the individualized PTV margin and CBCT-linac with 3 mm PTV margin. Center line indicates median, hinges depict 25th and 75th percentiles (inter-quartile range, IQR) and whiskers extend from the hinge to the largest/smallest value at maximally 1.5*IQR. Outlying data points (beyond end of the whiskers) are plotted individually. Hard constraints are plotted as solid horizontal lines, soft constraints as dashed lines. Asterisks depict significant differences in DVH parameters between MR-linac and both CBCT-linac plans (Mann-Whitney U-test (two-sided), n.s. $p \geq 0.05$, *$p < 0.05$, **$p < 0.01$, ***$p < 0.001$), with the lower bars indicating differences between MR-linac and CBCT-linac with the individualized PTV margins, and the upper bars indicating differences between MR-linac and CBCT-linac plans with 3 mm PTV margins. Note the different y-axis ranges of bowelbag and duodenum plots in subfigures I-N.

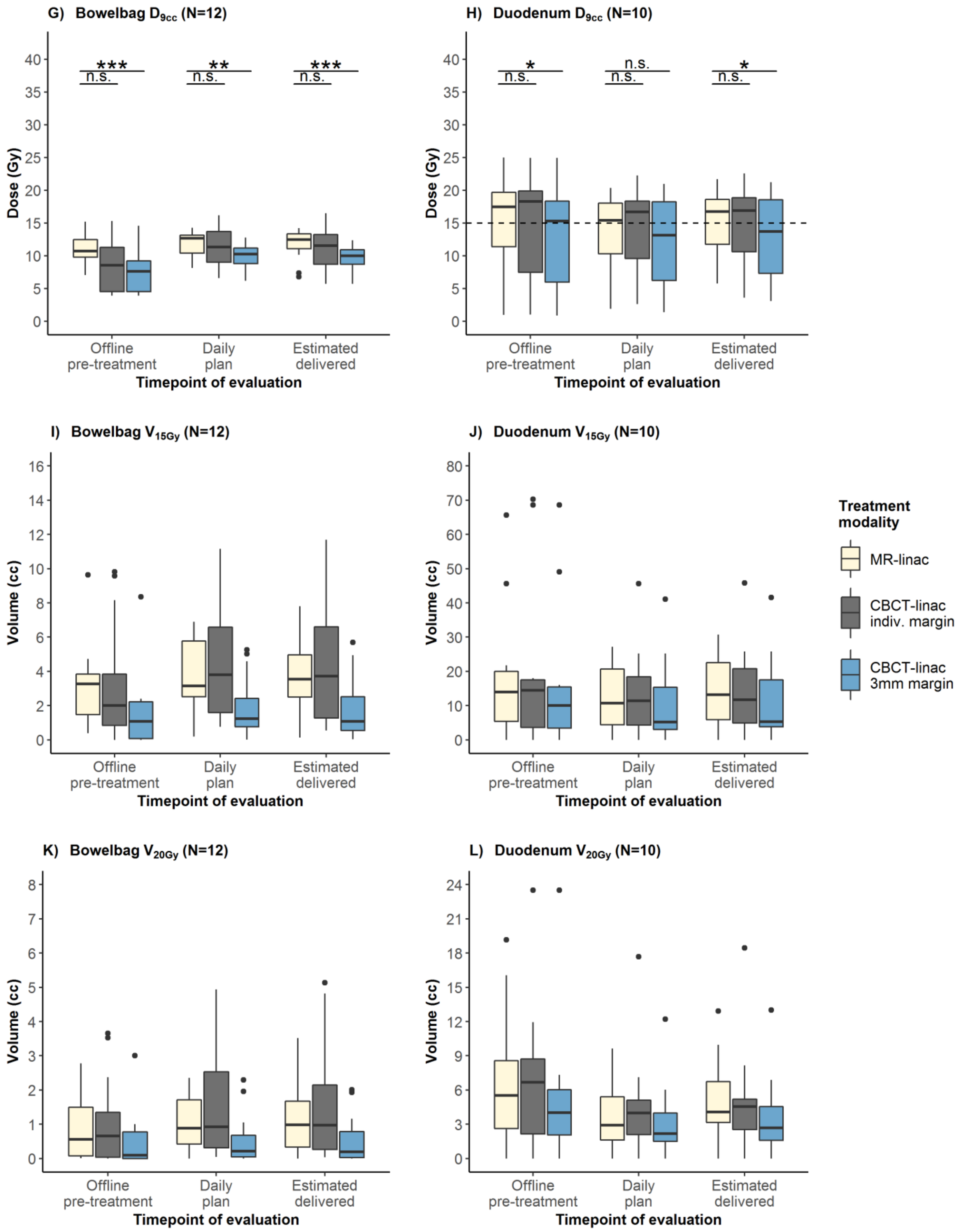

Supplementary Figure 2

Comparison of bowelbag and duodenum dose using MR-linac and CBCT-linac SBRT for lymph node oligometastases. *(continued)*

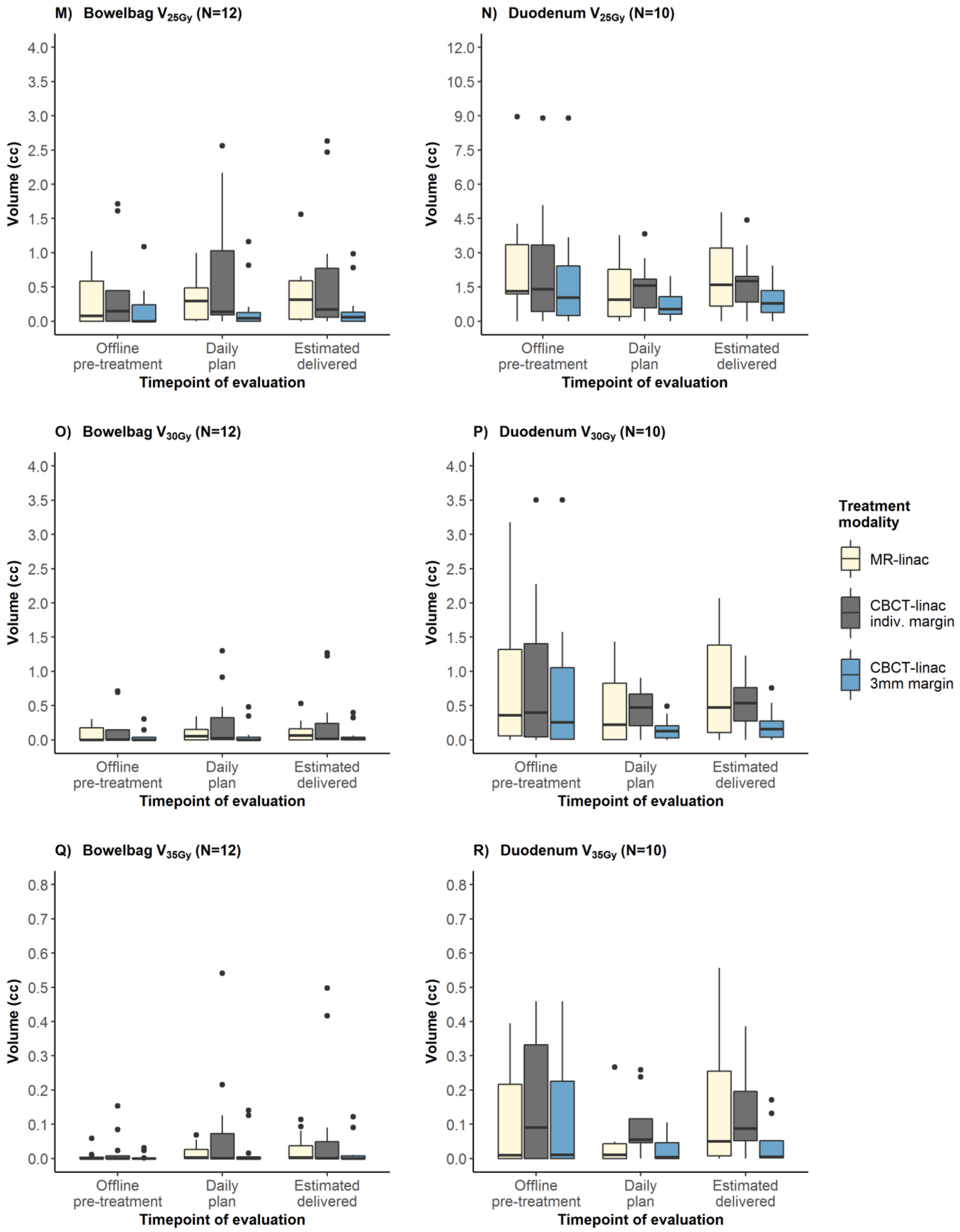

Supplementary Figure 2

Comparison of bowelbag and duodenum dose using MR-linac and CBCT-linac SBRT for lymph node oligometastases. *(continued)*

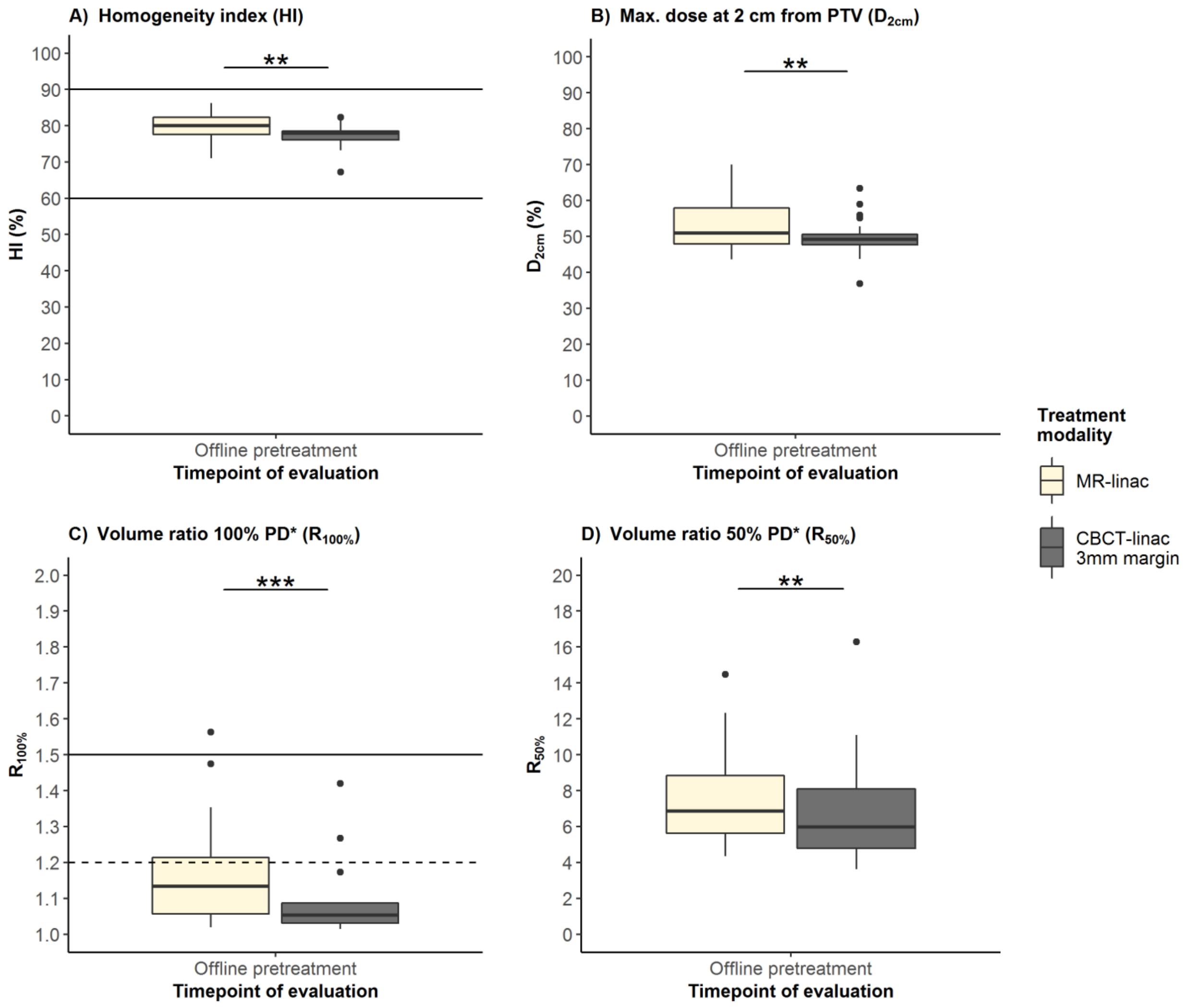

Supplementary Figure 3

Plan conformity/quality evaluation of MR-linac and CBCT-linac SBRT for lymph node oligometastases. A) homogeneity index (HI), B) maximum dose at 2 cm from PTV (D_{2cm}) relative to actual prescription dose (PD*, calculated as dose received by 95% of PTV), C) volume ratio of volume receiving 100% of PD* to PTV ($R_{100\%}$) and D) volume ratio at 50% of PD* ($R_{50\%}$). Results for offline pretreatment plans are shown for MR-linac and CBCT-linac, both with 3 mm PTV margins. In subfigure A, the solid lines indicate the acceptable values of HI, all outcomes were in the acceptable range. In subfigure C, values below the solid line are acceptable values and values below the dashed line are preferred values. Acceptable and preferred values are not plotted for D_{2cm} and $R_{50\%}$ as they depend on PTV volume (the target values are supplied as Supplementary Material: Table S2). D_{2cm} values were acceptable for both modalities for all patients. Asterisks depict significant differences in parameters between MR-linac and CBCT-linac plans (Mann-Whitney U-test (two-sided), n.s. $p \geq 0.05$, *$p < 0.05$, **$p < 0.01$, ***$p < 0.001$).
